# The mitochondrial outer membrane protein SYNJ2BP interacts with the cell adhesion molecule TMIGD1 and can recruit it to mitochondria

**DOI:** 10.1186/s12860-020-00274-1

**Published:** 2020-04-17

**Authors:** Christian Hartmann, Ysabel Alessa Schwietzer, Daniel Kummer, Nils Kirschnick, Esther Hoppe, Eva-Maria Thüring, Mark Glaesner-Ebnet, Frauke Brinkmann, Volker Gerke, Stefan Reuter, Masanori Nakayama, Klaus Ebnet

**Affiliations:** 1 Institute-Associated Research Group “Cell adhesion and cell polarity”, University of Münster, Von-Esmarch-Str. 56, 48149 Münster, Germany; 2 Institute of Medical Biochemistry, ZMBE, University of Münster, Von-Esmarch-Str. 56, 48149 Münster, Germany; 3 Interdisciplinary Clinical Research Center (IZKF), University of Münster, Von-Esmarch-Str. 56, 48149 Münster, Germany; 4 Department of Medicine D, Division of General Internal Medicine, Nephrology and Rheumatology, University Hospital of Münster, 48149 Münster, Germany; 5 Laboratory for Cell Polarity and Organogenesis, Max-Planck-Institute for Heart and Lung Research, 61231 Bad Nauheim, Germany; 6 Cells-in-Motion Cluster of Excellence (EXC 1003 - CiM), University of Münster, 48419 Münster, Germany

**Keywords:** Adhesion molecule, Cell-cell adhesion, JAM, Kidney epithelium, SYNJ2BP, TMIGD1

## Abstract

**Background:**

Transmembrane and immunoglobulin domain-containing protein 1 (TMIGD1) is a recently identified cell adhesion molecule which is predominantly expressed by epithelial cells of the intestine and the kidney. Its expression is downregulated in both colon and renal cancer suggesting a tumor suppressive activity. The function of TMIGD1 at the cellular level is largely unclear. Published work suggests a protective role of TMIGD1 during oxidative stress in kidney epithelial cells, but the underlying molecular mechanisms are unknown.

**Results:**

In this study, we address the subcellular localization of TMIGD1 in renal epithelial cells and identify a cytoplasmic scaffold protein as interaction partner of TMIGD1. We find that TMIGD1 localizes to different compartments in renal epithelial cells and that this localization is regulated by cell confluency. Whereas it localizes to mitochondria in subconfluent cells it is localized at cell-cell contacts in confluent cells. We find that cell-cell contact localization is regulated by N-glycosylation and that both the extracellular and the cytoplasmic domain contribute to this localization. We identify Synaptojanin 2-binding protein (SYNJ2BP), a PDZ domain-containing cytoplasmic protein, which localizes to both mitochondria and the plasma membrane, as interaction partner of TMIGD1. The interaction of TMIGD1 and SYNJ2BP is mediated by the PDZ domain of SYNJ2BP and the C-terminal PDZ domain-binding motif of TMIGD1. We also find that SYNJ2BP can actively recruit TMIGD1 to mitochondria providing a potential mechanism for the localization of TMIGD1 at mitochondria.

**Conclusions:**

This study describes TMIGD1 as an adhesion receptor that can localize to both mitochondria and cell-cell junctions in renal epithelial cells. It identifies SYNJ2BP as an interaction partner of TMIGD1 providing a potential mechanism underlying the localization of TMIGD1 at mitochondria. The study thus lays the basis for a better understanding of the molecular function of TMIGD1 during oxidative stress regulation.

## Background

Epithelial cells and endothelial cells are connected by cell adhesion receptors localized at intercellular junctions. These adhesion receptors are not only required to mediate physical cell cohesion but also to transmit signals and to mediate intercellular communication. The genes encoding cell adhesion receptors have been subjected to multiplication and diversification during evolution resulting in large families of cell-cell adhesion receptors, including the cadherin and the immunoglobulin superfamilies [1, 2]. The generation of large cell adhesion receptor superfamilies has been the basis of multicellularity and the development of higher organisms.

In most cases, cell-cell adhesion receptors interact with cytoplasmic adaptor proteins which either link the receptors to the cytoskeleton or to intracellular signalling pathways. The association with adaptor proteins is frequently mediated by sequence motifs like proline-rich motifs, FERM domain-binding motifs or PDZ domain-binding motifs, which interact with specific domains present in the adaptor proteins, such as SH3 domains, FERM domains or PDZ domains, respectively [3]. The promiscuous nature of many of the motif - domain interactions has further contributed to the pleiotropic functions of cell-cell adhesion receptors [4, 5].

Transmembrane and immunoglobulin domain-containing protein 1 (TMIGD1) is a member of the Ig-superfamily (IgSF) with similarity to the Junctional Adhesion Molecule (JAM) subfamily [6]. TMIGD1 contains two Ig-like domains of the C2-type, a single transmembrane region, and a short cytoplasmic domain of 21 amino acids (AA) that terminates in a canonical type I PDZ domain-binding motif [7]. It is expressed predominantly in the intestine and the kidney (https://www.proteinatlas.org/ENSG00000182271-TMIGD1/) [8]. Its expression is downregulated in tumours derived from intestine and kidney [8–11] as well as in inflamed intestinal tissues [12, 13], suggesting a tumour-suppressive function and a protective role during inflammation.

The function of TMIGD1 at the cellular level is largely unknown. Downregulation of TMIGD1 by RNA interference in cultured epithelial cells increases the cell’s susceptibility to oxidative stress, and its ectopic expression has a protective effect [14]. Also, reduced expression levels of TMIGD1 correlate with increased tissue damage after oxidative stress induced by ischemia and reperfusion in mice [14]. These findings suggest a protective role of TMIGD1 during oxidative stress, perhaps by influencing the metabolism of mitochondria which play a central role in the generation of reactive oxygen species [15]. The molecular mechanism through which TMIGD1 exerts its functions are completely unknown.

In this study, we identify Synaptojanin-2-binding protein (SYNJ2BP) as cytoplasmic binding partner of TMIGD1. SYNJ2BP has been described as a dual-location protein which localizes to the outer membrane of mitochondria [16, 17] as well as to the plasma membrane [18–20]. Our findings identify a cytoplasmic binding partner of TMIGD1 and provide the first mechanistic insights into the function of TMIGD1 in kidney epithelial cells.

## Results

### TMIGD1 is transported from the cytoplasm to the cell surface after deletion of the D1 Ig domain

**Fig. 1:**
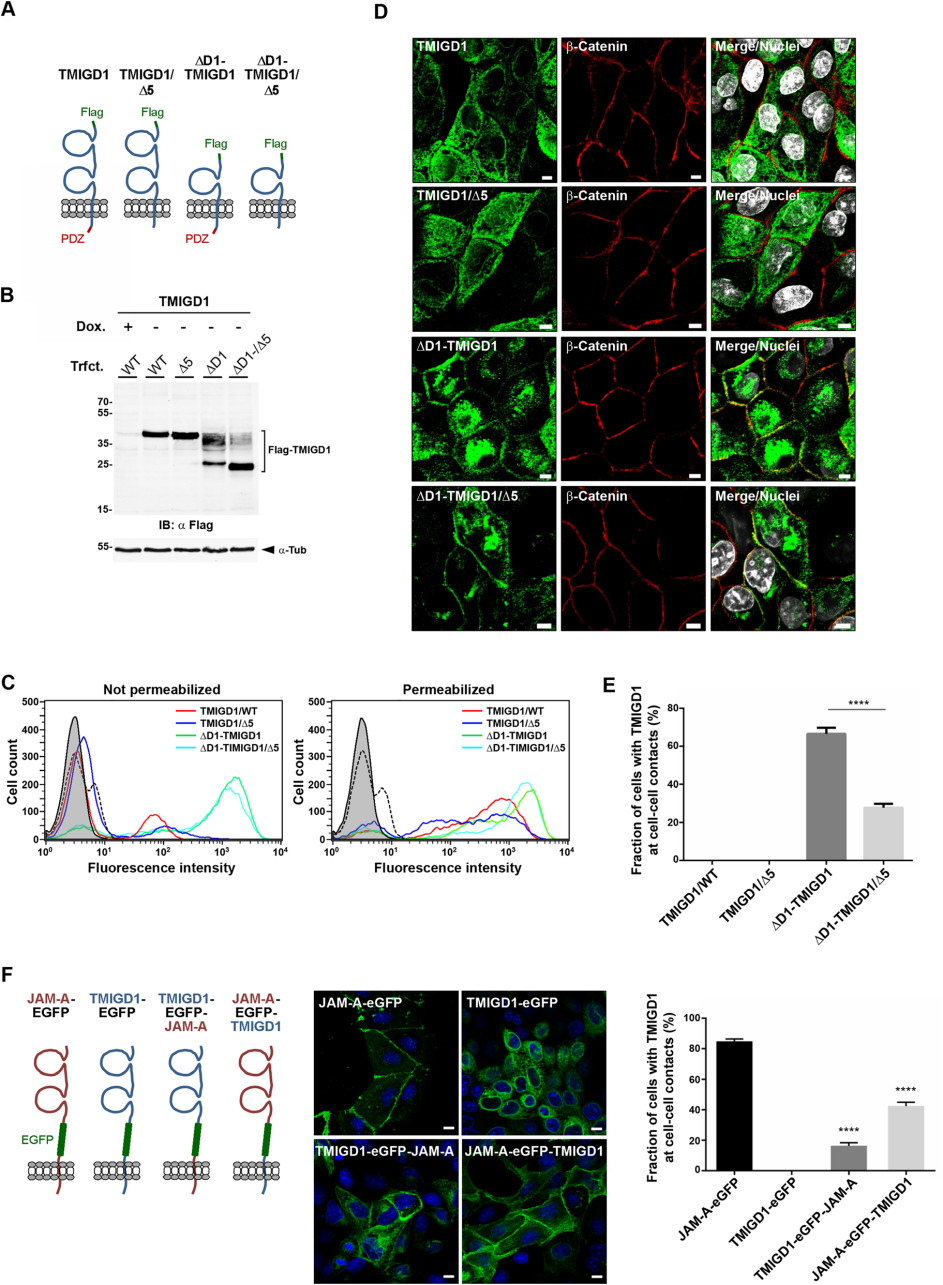
Ectopic TMIGD1 is localized in the cytoplasm of MDCKII cells but is efficiently transported to the cell surface after deletion of its D1 domain. **a** Schematic presentation of Flag-tagged TMIGD1 constructs expressed in MDCKII-TetOFF cells. The two ΔD1 mutants lack the membrane-distal Ig-like domain (D1 domain). The PDZ domain-binding motif (shown in red) is absent in the “Δ5” mutants. **b** MDCKII cell lines stably transfected with the TMIGD1 constructs depicted in (**a**) (TMIGD1/WT (WT), TMIGD1/Δ5 (Δ5), ΔD1-TMIGD1 (ΔD1), ΔD1-TMIGD1/Δ5 (ΔD1−/Δ5) were induced by doxycycline removal to express the transgenes and analyzed by Western blotting with antibodies against the Flag tag. **c** Flag-TMIGD1-expressing MDCKII-TetOFF cells were analyzed for cell surface localization of TMIGD1 constructs by flow cytometry using antibodies against the Flag tag. Cells were left untreated (Not permeabilized) to analyze cell surface proteins only, or were permeabilized by saponin treatment (Permeabilized) to analyze total protein levels. Note that the constructs with a complete extracellular domain (TMIGD1/WT, TMIGD1/Δ5) are predominantly localized in the cytoplasm, whereas constructs lacking the membrane-distal Ig-like domain (ΔD1-TMIGD1, ΔD1-TMIGD1/Δ5) are localized at the cell surface. Filled grey lines indicate unstained cells, dotted black lines indicate cells stained with secondary antibodies alone. **d** Immunofluorescence analysis of TMIGD1-expressing MDCKII cell lines depicted in panel (**a)**. Cells were stained with antibodies against the Flag tag and against β-catenin. Nuclei were stained with DAPI (pseudocoloured in white). Note that the constructs lacking the D1 Ig-like domain are localized at cell-cell contacts. Scale bars: 5 μm. **e** Quantification of cell-cell contact localization of TMIGD1 constructs. Cells were visually inspected for Flag signals at cell-cell contacts. TMIGD1 signals were rated as cell-cell contact-localized when a clear overlap with β-catenin signals at linear cell-cell contact sites was observed. Statistical analysis was performed using unpaired Student’s t-test. Data were obtained from three independent experiments and are presented as arithmetic means ± SEM; ****P < 0.0001. The differences in cell-cell contact localization between the two ΔD1-TMIGD1 constructs (ΔD1-TMIGD1, ΔD1-TMIGD1/Δ5) and each of the two constructs with entire extracellular domains (TMIGD1, TMIGD1/Δ5) are highly significant (*P* < 0.0001; not indicated by asterisks in the graph). **f** Cell-cell contact localization of TMIGD1 - JAM-A domain-swapping mutants. Left panel: Schematic presentation of domain swapping mutants. Middle panel: TMIGD1 - JAM-A swapping mutants shown in the left panel were transiently expressed in MDCKII cells. The subcellular localization was analyzed by fluorescence microscopy based on the EGFP signals. Right panel: Quantification of cell-cell contact localization of the TMIGD1 - JAM-A domain-swapping mutants. Cells were visually inspected for the localization of EGFP fluorescence signals at linear cell-cell contacts defined by β-catenin-positive signals (not shown). Statistical analyses were performed using unpaired Student’s t-test and display comparisons of the TMIGD1 swapping mutants with the TMIGD1 full length protein (TMIGD1-EGFP). Data were obtained from three independent experiments and are presented as arithmetic means ± SEM; ***P < 0.001

Given the high similarity of TMIGD1 to members of the Junctional Adhesion Molecule (JAM) family [6], and its predominant expression by kidney-derived epithelial cells [8, 14], we aimed to characterize the subcellular localization of TMIGD1 in kidney epithelial cells. To this, we expressed TMIGD1 constructs in MDCKII cells, a canine kidney epithelial cell line derived from distal kidney tubules [21]. Stable MDCKII-TetOFF cell lines were generated which allow the expression of either wildtype TMIGD1 (TMIGD1/WT) or TMIGD1 lacking the PDZ domain-binding motif (TMIGD1/Δ5) under a doxycycline-regulated promoter, as described previously for JAM-A [22]. Since studies with other JAM family members indicated a critical role for the membrane-distal Ig-like domain for their enrichment at cell-cell contacts [23], we also generated cell lines expressing a TMIGD1 construct lacking the membrane-distal Ig-like domain (ΔD1-TMIGD1) as well as a TMIGD1 construct that lacks both the membrane-distal Ig-like domain and the PDZ domain-binding motif (ΔD1-TMIGD1/Δ5) (Fig. 1a). All constructs were expressed at similar levels after removal of doxycycline from the culture medium, as analyzed by Western blotting (Fig. 1b). Surprisingly, both TMIGD1/WT and TMIGD1/Δ5 were not detectable at the cell surface when analyzed by flow cytometry, whereas both ΔD1-TMIGD1 constructs were localized at the cell surface (Fig. 1b). Immunofluorescence stainings confirmed an exclusive localization of TMIGD1/WT and TMIGD1/Δ5 in cytoplasmic compartments and a localization of ΔD1-TMIGD1 and ΔD1-TMIGD1/Δ5 both in cytoplasmic compartments and at cell-cell junctions (Fig. 1d). These observations thus suggested that the D1 Ig-like domain of TMIGD1 prevents its transport to the cell surface in MDCKII cells. Quantification of cell-cell contacts positive for ΔD1-TMIGD1 and ΔD1-TMIGD1/Δ5 indicated that the number of ΔD1-TMIGD1/Δ5-positive contacts was significantly lower than the number of ΔD1-TMIGD1-positive contacts (Fig. 1e), strongly suggesting that a PDZ domain-mediated interaction is required for the stable localization of this construct at cell-cell junctions.

To further address the contribution of the extracellular and the cytoplasmic domain of TMIGD1 to its cell-cell contact localization, we performed localization studies using TMIGD1 - JAM-A swapping mutants. JAM-A has an overall organization similar to TMIGD1 with two Ig-like domains, a single transmembrane spanning region and a short cytoplasmic domain, but in contrast to TMIGD1, JAM-A is constitutively localized at cell-cell contacts when expressed in MDCK cells [22]. In one construct we replaced the transmembrane and cytoplasmic domains of TMIGD1 (TMIGD1 - EGFP - JAM-A), in the second construct we replaced the extracellular domain of TMIGD1 (JAM-A - EGFP - TMIGD1) (Fig. 1f, schematics). The TMIGD1 control construct (TMIGD1 - EGFP - TMIGD1) did not localize to cell-cell contacts (Fig. 1f), as observed for the Flag-tagged TMIGD1 constructs (Fig. 1c, d). Replacing the transmembrane and cytoplasmic regions slightly increased the fraction of cell contact-localized TMIGD1 constructs to approximately 20% of transfected cells (Fig. 1f). Replacing the extracellular domain of TMIGD1 increased the fraction of cell contact-localized TMIGD1 constructs to approximately 40% of transfected cells (Fig. 1f). These findings suggest that both the extracellular and the transmembrane/cytoplasmic regions of TMIGD1 contribute to its retention in the cytoplasm. Retention in the cytoplasm may be regulated by glycosylation of the extracellular domain as well as by interaction of the cytoplasmic domain with scaffolding proteins, as shown for the glutamate transporter GLAST [24].

### TMIGD1 is localized at cell-cell junctions and in the cytoplasm of kidney epithelial cells and is preferentially expressed by epithelial cells of proximal tubules

**Fig. 2:**
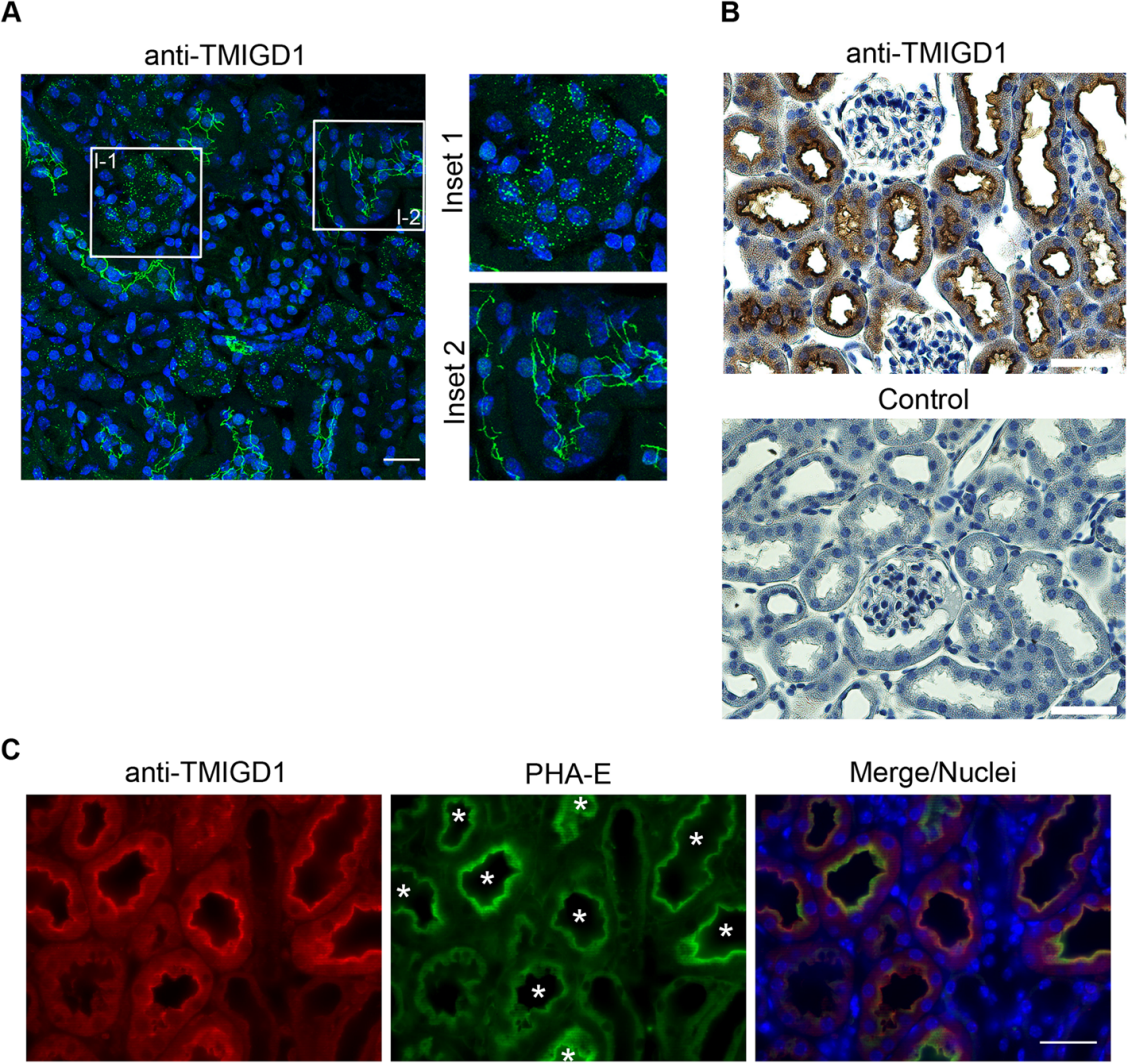
TMIGD1 is expressed by renal epithelial cells with distinct subcellular locations. **a** Immunofluorescence analysis of murine kidney sections with TMIGD1 antibodies (#HPA021946). The insets (Inset 1, Inset 2) show magnifications of the areas demarcated with rectangles (I-1, I-2) in the left panel. Note that TMIGD1 is enriched at cell-cell contacts in some tubular epithelial cells but is localized in the cytoplasm in others. Scale bar: 100 μm. **b** Immunohistochemistry on murine kidney sections with antibodies against TMIGD1 (#HPA021946). Control stainings (bottom panel) were performed in the absence of the primary antibodies. Note that TMIGD1 is expressed by tubular epithelial cells but is absent from glomerular cells. Scale bars: 50 μm. **c** Immunofluorescence analysis of murine kidney sections. Kidney sections were stained with FITC-conjugated PHA-E lectin and with anti-TMIGD1 antibodies (#HPA021946). Note that all TMIGD1-positive structures are positive for PHA-E (marked by asterisks) suggesting preferential expression of TMIGD1 by epithelial cells of proximal tubules. Scale bar: 100 μm

Besides the small intestine, TMIGD1 is predominantly expressed by epithelial cells in the kidney [8, 9]. Immunohistochemistry of kidney sections revealed a localization of TMIGD1 both at cell-cell contacts and in cytoplasmic compartments of renal tubular epithelial cells but a complete absence of TMIGD1 expression in glomerular podocytes (Fig. 2a, b). These observations are in line with previous findings showing expression of TMIGD1 in kidney tubules [14]. Co-stainings of TMIGD1 with PHA-E, a marker for proximal kidney tubules [25], indicated that TMIGD1 is predominantly expressed by epithelial cells in proximal tubules (Fig. 2c). Together, these observations show that TMIGD1 is differently expressed by epithelial cells in different kidney compartments with a complete absence in glomerular podocytes. They also suggest that the subcellular localization of TMIGD1 might be different in epithelial cells derived from these different compartments, such as proximal and distal kidney tubules, which could provide a possible explanation of the exclusive localization of ectopically expressed TMIGD1 in the cytoplasm of MDCK cells (Fig. 1c, d), which are derived from distal kidney tubules [21].

### N-glycosylation of ΔD1-TMIGD1 regulates its cell surface localization

**Fig. 3:**
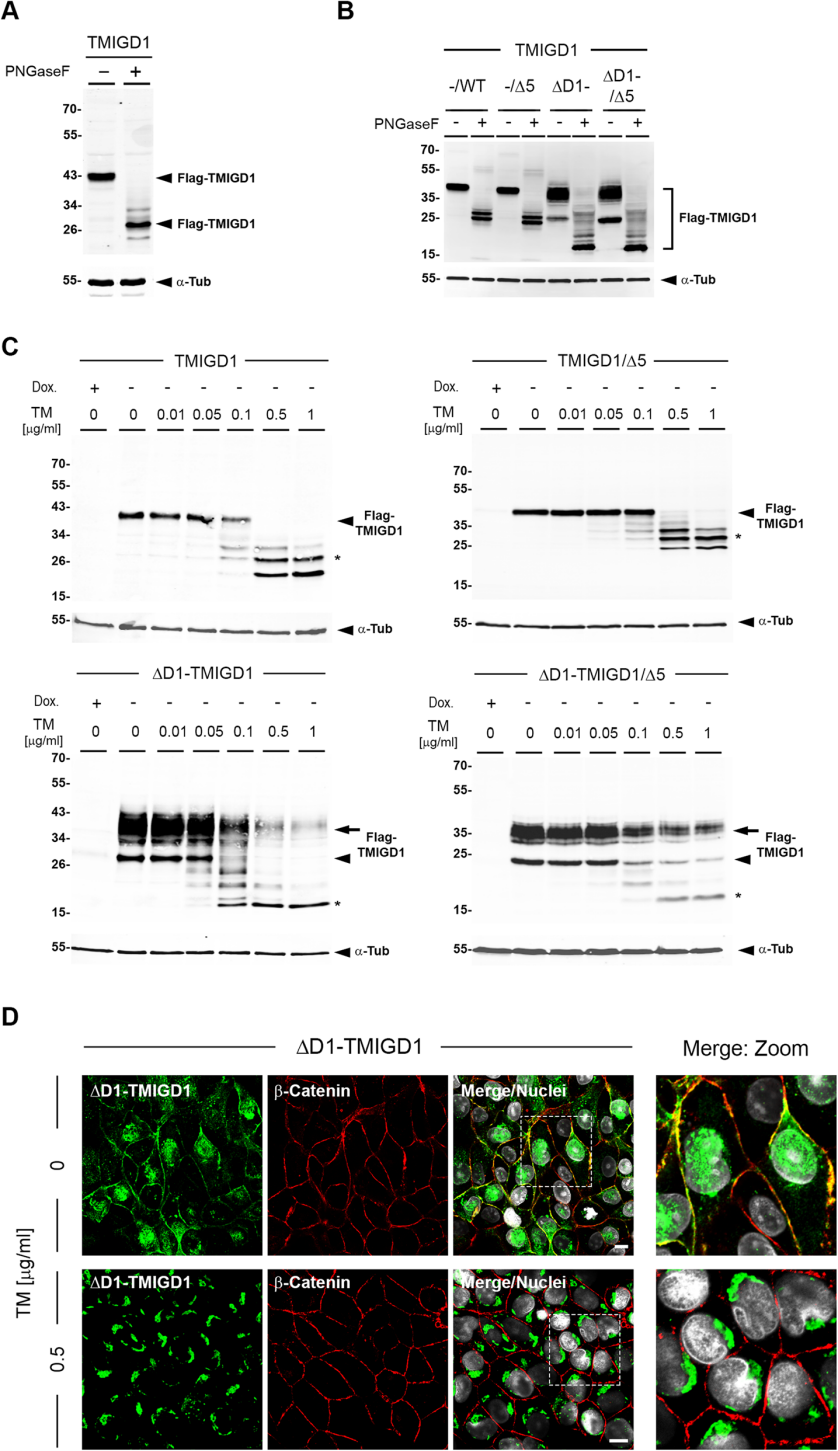
N-glycosylation regulates TMIGD1 localization at cell-cell contacts. **a** Lysates obtained from Flag-TMIGD1-expressing HEK293T cells were incubated with PNGaseF, separated by SDS-PAGE and analyzed by Western blotting with antibodies against the Flag tag. Note that the electrophoretic mobility of TMIGD1 shifts to a M_r_ that corresponds to the M_r_ expected for the unglycosylated protein, indicating that TMIGD1 is decorated exclusively with N-linked carbohydrates. **b** Lysates obtained from MDCKII cell lines expressing Flag-tagged TMIGD1 constructs (TMIGD1/WT (−/WT), TMIGD1/Δ5 (−/Δ5), ΔD1-TMIGD1 (ΔD1-), ΔD1-TMIGD1/Δ5 (ΔD1−/Δ5) were incubated with PNGaseF and analyzed by Western blotting with antibodies against the Flag tag. The electrophoretic mobilities of all TMIGD1 constructs shift to M_r_ that correspond to the M_r_ expected for the unglycosylated proteins. Note that the absence of the D1 domain (ΔD1, ΔD1−/Δ5 constructs) results in a protein species with a M_r_ similar to that of the full length proteins (/WT, /Δ5, respectively). These protein species most likely result from hyperglycosylation in the absence of the D1 Ig-like domain. **c** MDCKII cell lines described in (**b**) were incubated for 20 h with various concentrations of tunicamycin. Lysates were analyzed by Western blotting with antibodies against the Flag tag. Arrowheads indicate the TMIGD1 isoforms in the absence of tunicamycin treatment, asterisks indicate the TMIGD1 isoforms expected from the unmodified proteins. Protein bands above and below the protein bands marked by asterisks most likely represent incompletely deglycosylated protein species and degradation products due to the lack of glycosylation, respectively. **d** ΔD1-TMIGD1-expressing MDCKII cells were fixed with methanol and double-stained with antibodies against the Flag tag and against β-catenin. Flag-ΔD1-TMIGD1 is completely absent from cell-cell contacts after treatment with 0.5 μg/ml tunicamycin. Abbreviations: Dox., doxycycline, TM, tunicamycin, α-tub, α-tubulin. Scale bars: 10 μm

Cell surface localization of integral membrane proteins is regulated by glycosylation [26]. TMIGD1 contains five potential N-linked glycosylation sites (consensus: N – X – S/T/C, X = any AA except P) in the EC domain and has been described to be N-glycosylated [14]. We therefore analyzed the glycosylation of TMIGD1 in HEK293T cells and MDCKII cells. Treatment of cell lysates obtained from TMIGD1-expressing HEK293T cells with PNGaseF, an endoglycosidase that efficiently removes almost all N-linked oligosaccharides from glycoproteins, resulted in a shift of the M_r_ of TMIGD1 from approximately 43 kDa to approx. Twenty-seven kDa, which corresponds to the predicted M_r_ of the unglycosylated Flag-tagged protein (27.6 kDa) (Fig. 3a). Similarly, PNGaseF treatment of lysates obtained from MDCKII cells expressing the various TMIGD1 constructs depicted in Fig. 1a resulted in a shift to the M_r_ expected from the unmodified proteins in all constructs (Fig. 3b) indicating that all constructs are N-glycosylated. Of note, the TMIGD1 constructs lacking the D1 Ig-like domain (ΔD1-TMIGD1, ΔD1-TMIGD1/Δ5) showed an abundant protein species with a M_r_ similar to the constructs containing both Ig-like domains (Fig. 3b). PNGaseF treatment completely abolished this high M_r_ species, indicating that this protein species results from hyperglycosylation. The increased glycosylation of TMIGD1 in the absence of the D1 Ig-like domain might result from an unmasking of N-linked glycosylation sites that are not accessible for glycosylation in the full length protein.

To further address the glycosylation of the TMIGD1 constructs in MDCKII cells, cells were treated for 20 h with tunicamycin, an inhibitor of N-glycosidic linkage formation in glycoprotein synthesis, and analyzed by Western blotting with antibodies against Flag-tagged TMIGD1. Similar to the treatment of lysates with PNGaseF, incubation of cells with tunicamycin resulted in a dose-dependent shift of the M_r_ of all TMIGD1 constructs to lower M_r_ species (Fig. 3c). The high M_r_ species observed for the two ΔD1 constructs were strongly reduced in the presence of 1 μg/ml tunicamycin confirming that these species result from N-glycosylation. To test if this hyperglycosylation is involved in the cell surface and cell-cell contact localization of ΔD1-TMIGD1, we analyzed its localization by immunocytochemistry. In the presence of tunicamycin, ΔD1-TMIGD1 was completely absent from intercellular junctions but was present in the cytoplasm, most likely in the Golgi apparatus (Fig. 3d, Suppl. Fig. 1). Together, these observations suggest that the junctional localization of TMIGD1 is regulated by N-glycosylation and that one (or more) N-glycosylation sites required for junctional localization might be masked in the full length protein.

### TMIGD1 is localized in mitochondria in subconfluent cells and is recruited to intercellular junctions during maturation in HK-2 kidney epithelial cells

**Fig. 4:**
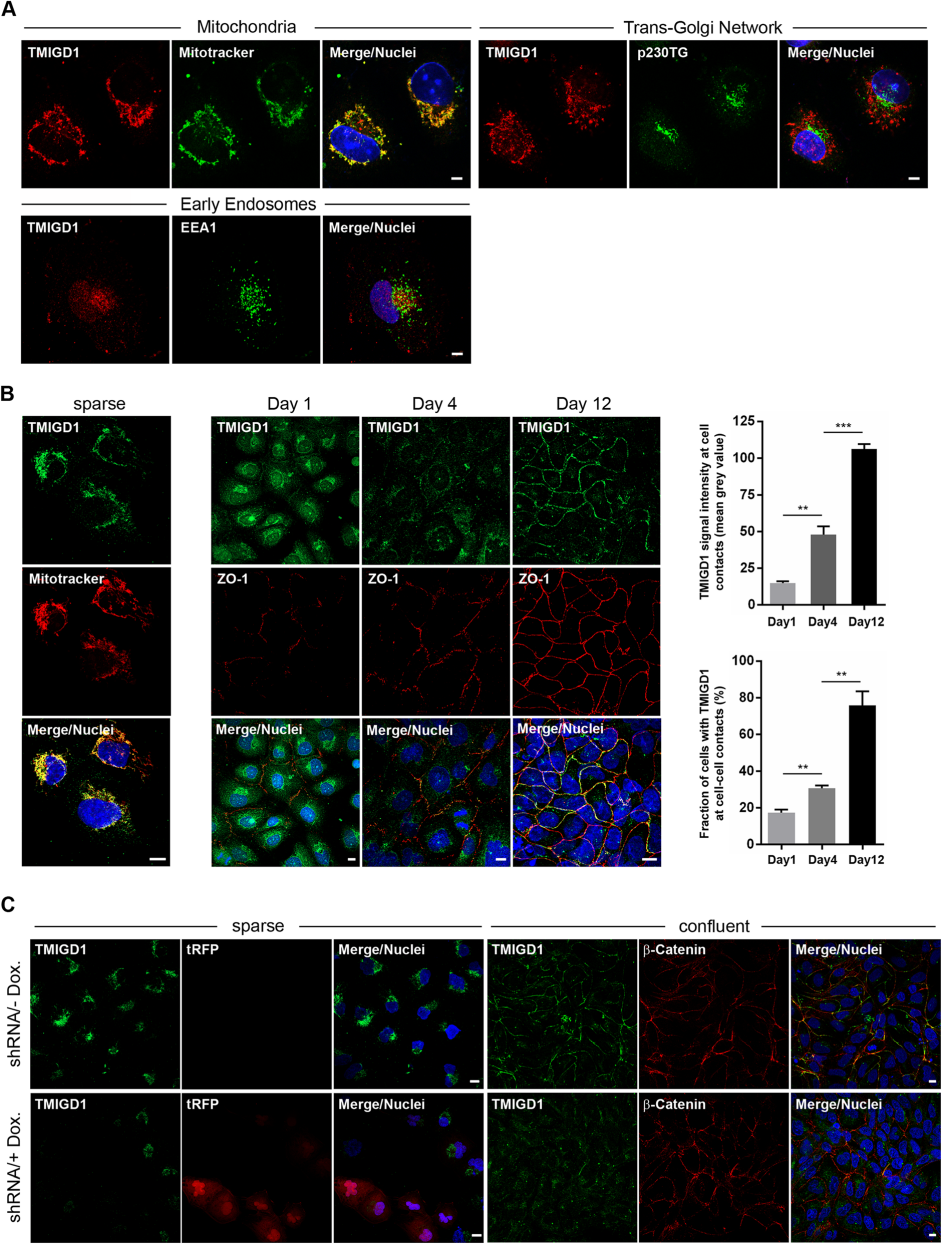
TMIGD1 localizes to mitochondria and cell-cell contacts in HK-2 cells. **a** Sparsely grown HK-2 cells were fixed and stained with polyclonal antibodies against TMIGD1 (#HPA021946) and markers for mitochondria (MitoTracker), the trans-Golgi network (p230TG) and early endosomes (EEA1). Note that TMIGD1 localizes specifically to mitochondria. Scale bars: 5 μm. **b** HK-2 cells were grown as single cells or were grown and maintained at confluency for different periods of time, as indicated. Cells were fixed and stained with antibodies against TMIGD1 (#HPA021946) and MitoTracker (sparsely grown cells), or with antibodies against TMIGD1 (#HPA021946) and the cell-cell contact marker ZO-1 (cells grown to confluency). Right panels: Quantification of TMIGD1 signal intensities at cell-cell contacts (top bar graph) and of fraction of cells with TMIGD1-positive cell-cell contacts at different days of confluency (bottom bar graph). Quantification of TMIGD1 signal intensities at cell-cell contacts (top bar graph) was performed as described in the Methods section. Statistical analysis was performed using unpaired Student’s t-test. Data were obtained from three independent experiments and are presented as arithmetic means ± SEM; **P < 0.01, ***P < 0.001. Scale bars: 10 μm. **c** HK-2 cells stably transfected with the pInducer10-mir-RUP-PheS plasmid vector that allows expression of both a TMIGD1-specific shRNA and turboRFP (tRFP) under a doxycycline-regulated promoter were grown to low confluency (sparse) or high confluency (confluent), fixed with PFA and methanol, respectively, and analyzed by fluorescence microscopy with antibodies against TMIGD1 (#HPA021946) and the cell contact marker β-catenin, as indicated. In sparsely grown cells, the tRFP signal encoded by the pInducer10 vector was used as marker for shRNA-expressing cells. Note that the pInducer10 vector is based on a TetON system, i.e. shRNA expression is induced by doxycycline. Scale bars: 10 μm

Given the preferential cytoplasmic localization of ectopically expressed TMIGD1 in distal tubule-derived kidney MDCKII epithelial cells (Fig. 1) and the different localization and expression of TMIGD1 in proximal vs distal kidney tubules (Fig. 2), we analyzed the localization of endogenous TMIGD1 in HK-2 cells, a human renal epithelial cell line derived from proximal tubules [27]. At low cell density TMIGD1 was localized in a cytoplasmic compartment. Co-stainings with markers specific for mitochondria (MitoTracker), the Golgi apparatus (p230TG), or early endosomes (EEA-1), revealed localization of TMIGD1 in mitochondria but not in the Golgi apparatus or in early endosomes (Fig. 4a). When cells were grown to higher densities, TMIGD1 gradually localized at cell-cell contacts with the highest number of TMIGD1-positive cells and strongest TMIGD1 signal intensity being observed at 12 days of confluency (Fig. 4b). These observations indicated that TMIGD1 can localize to both mitochondria and to intercellular junctions, and that the localization at cell-cell junctions is regulated by cell density.

To confirm the specificity of the antibody-generated signals in mitochondria of sparse cells and at cell-cell junctions of confluent cells, we transfected HK-2 cells with a vector that allows inducible expression of a TMIGD1 shRNA and analyzed the cells under sparse and confluent growth conditions. Both the mitochondrial signal and the cell junction-associated signal were largely abolished after shRNA-mediated knockdown of TMIGD1 (Fig. 4c) confirming TMIGD1 as a dual-location protein.

### TMIGD1 interacts with SYNJ2BP

**Fig. 5:**
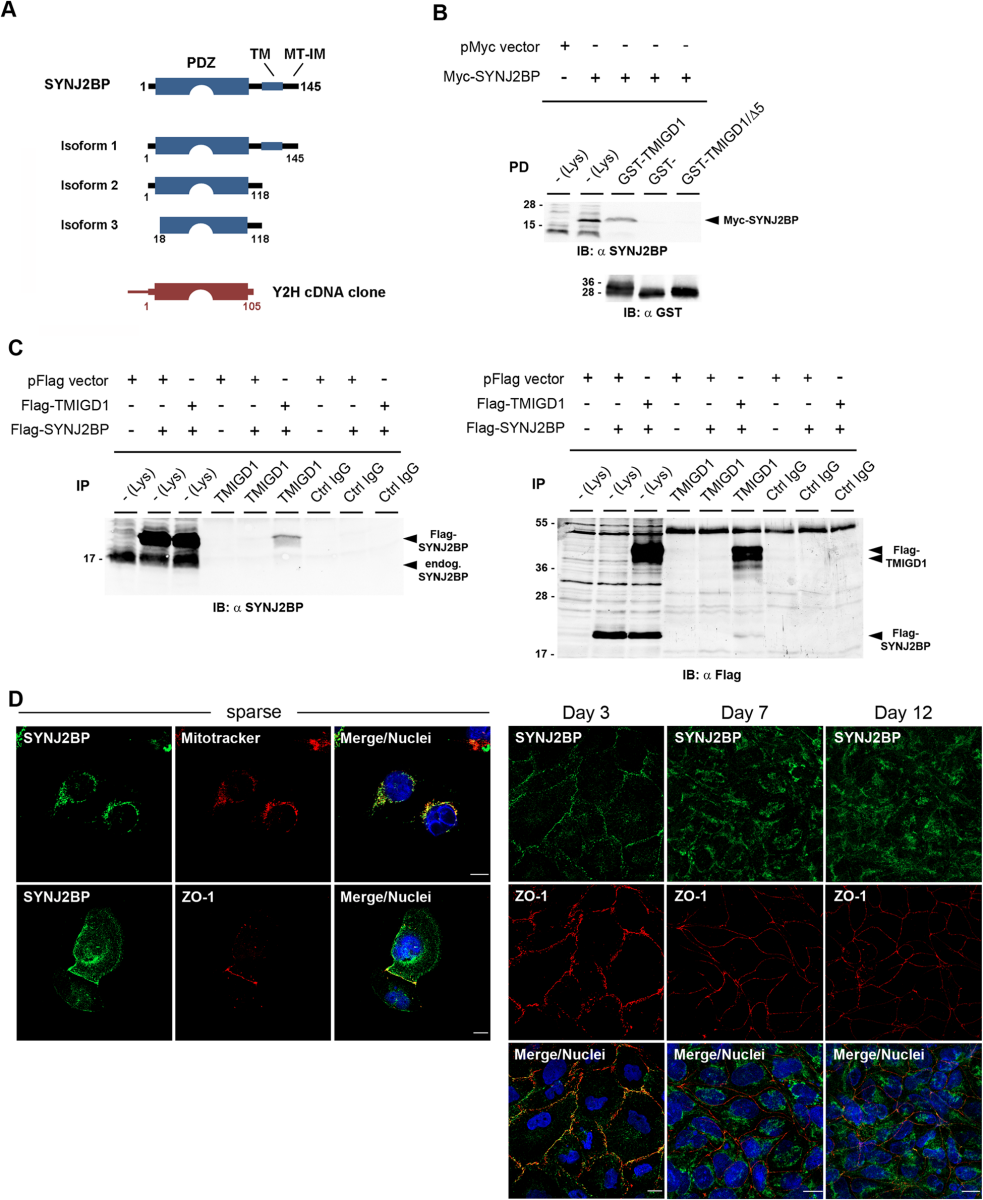
TMIGD1 interacts with SYNJ2BP. **a** Schematic presentation of murine Synj2bp isoforms. The TMIGD1-interacting fragment isolated from the yeast-two hybrid library (indicated in red) encodes AA 1–105 of Synj2bp and encompasses the entire PDZ domain (AA 13–100). Abbreviations: TM, transmembrane, MT-IM, mitochondrial intermembrane (**b**) TMIGD1 interacts with SYNJ2BP through its PDZ domain-binding motif. Lysates of SYNJ2BP-transfected HEK293T cells were incubated with GST-fusion proteins containing the entire cytoplasmic domain of TMIGD1 (GST-TMIGD1) or the cytoplasmic domain lacking the PDZ domain-binding motif (GST-TMIGD1/Δ5) or with GST alone (GST-). Precipitates obtained with GST fusion proteins were immunoblotted with anti-SYNJ2BP antibodies (Sigma #HPA000866, top panel, 90% of precipitates) or with anti-GST antibodies (bottom panel, 10% of precipitates). SYNJ2BP precipitated with GST-TMIGD1 but not with GST-TMIGD1/Δ5. Abbreviations: IB, immunoblotting; Lys, lysate. **c** SYNJ2BP co-immunoprecipitates with TMIGD1. Immunoprecipitates obtained with anti-TMIGD1 polyclonal antibodies (Affi1611) from HEK293T cells transfected with empty vector (pFlag vector), Flag-TMIGD1 and/or Flag-SYNJ2BP as indicated were immunoblotted with anti-SYNJ2BP antibodies (Sigma #HPA000866, left panel, 90% of precipitates) or with anti-Flag antibodies (right panel, 10% of precipitates). Note that SYNJ2BP is present in TMIGD1 immunoprecipitates. Abbreviations: endog., endogenous; IB, immunoblotting; IP, immunoprecipitation; Lys, lysate. **d** SYNJ2BP localizes at mitochondria in sparsely seeded cells but to cell-cell contacts in confluent cells. HK-2 cells were either sparsely seeded or grown to confluency for 3d, 7d, or 12 d as indicated, then fixed and stained for SYNJ2BP and either Mitotracker or ZO-1 as indicated. Scale bars: 10 μm

To identify molecules that interact with TMIGD1 and possibly regulate its localization in mitochondria, we performed a yeast-two hybrid screen using the cytoplasmic tail of TMIGD1 as bait and a murine embryonic cDNA library [28, 29]. We isolated a cDNA fragment that covered nucleotides 78 to 448 of the murine SYNJ2BP cDNA and that encompassed AA 1–105 of the murine SYNJ2BP isoform 1 [30]. This clone encodes for the entire PDZ domain of SYNJ2BP (AA13–100) with only a few additional AA flanking the PDZ domain at both termini (Fig. 5a), which strongly suggests a PDZ domain-dependent interaction between TMIGD1 and SYNJ2BP. To verify this interaction we performed GST-pulldown experiments. GST-TMIGD1 cytoplasmic domain fusion proteins immobilized on GSH-sepharose were incubated with lysates obtained from SYNJ2BP-transfected HEK293T cells. SYNJ2BP was precipitated with the wildtype cytoplasmic domain of TMIGD1 but not with a deletion mutant lacking the PDZ domain-binding motif (TMIGD1/Δ5, Fig. 5b). These findings confirmed that TMIGD1 interacts with SYNJ2BP and indicated that this interaction requires the PDZ domain-binding motif of TMIGD1. To address an interaction between TMIGD1 and SYNJ2BP in cells, we performed co-immunoprecipitation (CoIP) experiments. SYNJ2BP co-immunoprecipitated with TMIGD1 from transfected HEK293T cells (Fig. 5c), indicating that TMIGD1 and SYNJ2BP interact in cells. Together, these observations identify SYNJ2BP as binding partner of TMIGD1 that interacts with TMIGD1 through a direct PDZ domain-dependent interaction.

Since SYNJ2BP is described as a protein that localizes to mitochondria and to the plasma membrane [16–20], we analyzed its localization in HK-2 cells grown to different densities. Similar to what we observed for TMIGD1, SYNJ2BP localized predominantly to mitochondria in sparse cells but was localized at cell-cell contacts when grown to confluency (Fig. 5d). When cells were grown for 7 or 12 days at confluency, SYNJ2BP was no longer present at cell-cell contacts but localized predominantly in the cytoplasm (Fig. 5d). These observations indicate that similar to TMIGD1, SYNJ2BP shows a dynamic localization at cytoplasmic compartments and at cell-cell junctions in a confluency-dependent manner.

### SYNJ2BP can recruit TMIGD1 to mitochondria

**Fig. 6:**
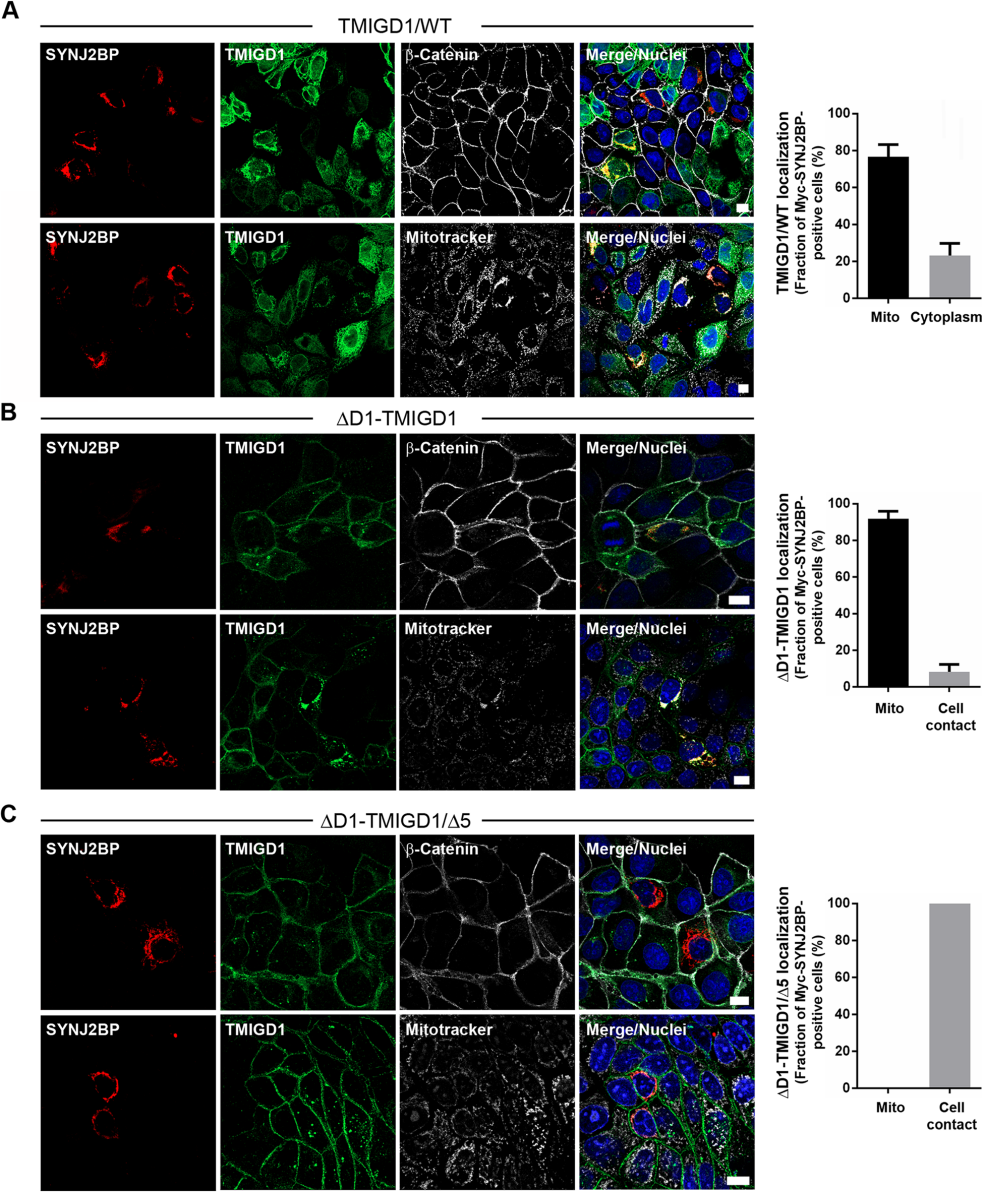
Ectopic Myc-SYNJ2BP recruits TMIGD1 to mitochondria in a PDZ domain-dependent manner. MDCKII-TetOFF cells stably expressing either Flag-TMIGD1 WT (**a**), Flag-ΔD1-TMIGD1 (**b**), or Flag-ΔD1-TMIGD1/Δ5 (**c**) were transiently transfected with Myc-SYNJ2BP. Cells were stained with antibodies against Myc-SYNJ2BP (anti Myc, SantaCruz #sc-789G), Flag-TMIGD1 (anti Flag, Sigma #F7425), and either endogenous β-catenin to visualize cell-cell contacts or with mitotracker to visualize mitochondria, as indicated. The bar graphs in each panel show quantifications of Flag-TMIGD1 localization in Myc-SYNJ2BP-expressing cells (TMIGD1 co-localization with Myc-SYNJ2BP in mitochondria vs TMIGD1 localization in the cytoplasm (panel **a**), or TMIGD1 co-localization with Myc-SYNJ2BP in mitochondria vs TMIGD1 localization at β-catenin-positive cell contacts (ΔD1-TMIGD1 and ΔD1-TMIGD1/Δ5, panels **b** and **c**). Data are indicated as the fraction of Myc-SYNJ2BP-positive cells that are positive for TMIGD1 at mitochondria vs cytoplasm (**a**) or cell contacts (**b**, **c**). Data were obtained from at least three independent experiments with at least 50 cells analyzed. Error bars indicate standard deviations between experiments. Scale bars: 10 μm

To establish a functional interaction between TMIGD1 and SYNJ2BP, we ectopically expressed Myc-tagged SYNJ2BP in TMIGD1-transfected MDCKII-TetOFF cell lines (depicted in Fig. 1) and analyzed the localization of TMIGD1 by immunofluorescence microscopy. Transfected Myc-SYNJ2BP localized exclusively to mitochondria and was absent from cell-cell contacts in all cell lines tested (Fig. 6). TMIGD1/WT was recruited from cytoplasmic compartments to mitochondria in approximately 75% of Myc-SYNJ2BP-expressing cells (Fig. 6a), suggesting that SYNJ2BP can recruit TMIGD1 to mitochondria. The ΔD1-TMIGD1 construct, which is localized at cell-cell contacts in the absence of ectopic SYNJ2BP (Fig. 1c, e; Fig. 3d), was recruited to mitochondria in more than 90% of Myc-SYNJ2BP-expressing cells (Fig. 6b). Deletion of the PDZ domain-binding motif in ΔD1-TMIGD1 (ΔD1-TMIGD1/Δ5) resulted in a complete retention of this construct at cell-cell junctions (Fig. 6c). These findings indicate that SYNJ2BP can recruit TMIGD1 to mitochondria, and that this recruitment is mediated by the PDZ domain-binding motif of TMIGD1.

## Discussion

In the present study, we characterize the subcellular localization of TMIGD1, a cell adhesion receptor that is expressed in epithelial cells of the intestine and the kidney [8, 9, 14]. In MDCKII cells, a kidney epithelial cell line derived from distal tubules [21], TMIGD1 is localized predominantly in the cytoplasm. Deletion of the membrane-distal Ig-like domain of TMIGD1 results in transport to the cell surface and cell-cell contact localization. The localization of this construct at cell-cell contacts correlates with increased N-linked glycosylation, suggesting that this localization can be regulated by N-glycosylation. Experiments with domain swapping mutants indicate that both the extracellular domain and the cytoplasmic domain of TMIGD1 contribute to its localization at cell-cell contacts. Surprisingly, in HK-2 cells, a kidney epithelial cell line derived from proximal tubules [27], TMIGD1 localizes to mitochondria when cells are grown at low confluency but is recruited to cell-cell contacts with increasing cell density. Our findings thus identify TMIGD1 as an adhesion receptor with different subcellular localization and indicate that its subcellular localization can be regulated by cell density.

We identified SYNJ2BP as an interaction partner of TMIGD1, providing a possible explanation for the localization of TMIGD1 in mitochondria in HK-2 cells. The SYNJ2BP cDNA clone isolated from the murine Y2H library comprised AA 1–105 of SYNJ2BP, which represents almost exclusively the PDZ domain of SYNJ2BP (AA 13–100). GST-pulldown experiments indicated that the PDZ domain-binding motif of TMIGD1 (−SETAL) is required for the interaction with SYNJ2BP. In addition, ectopically expressed SYNJ2BP recruited ΔD1-TMIGD1 efficiently to mitochondria whereas the same construct lacking the PDZ binding motif (ΔD1-TMIGD1/Δ5) was retained at cell-cell contacts (Fig. 6). Our observations therefore strongly suggest a direct and PDZ domain-dependent interaction between TMIGD1 and SYNJ2BP.

SYNJ2BP, a.k.a. as mitochondrial outer membrane protein 25 (OMP25) or activin receptor-interacting protein 2 (Arip2), has originally been described as a mitochondria-associated protein of 145 AA [16]. SYNJ2BP consists of a PDZ domain (AA 13–100) that localizes in the cytoplasm, a transmembrane domain (TMD, AA 118–138) through which the protein is inserted in the outer mitochondrial membrane, and a short C-terminus (AA 139–145) that extends into the mitochondrial intermembrane region [16, 17] (Fig. 5a). Two out of three identified splice variants lack the TMD and thus are expected to localize exclusively in the cytoplasm [30]. Interestingly, SYNJ2BP has been found to interact with the cytoplasmic domain of activin type II receptors (ActRII) to regulate their endocytosis [18], as well as with the cytoplasmic domains of the Notch ligands Delta like protein (DLL) 1 and DLL4 to increase protein stability and Notch signalling [19]. Together, these observations demonstrate that SYNJ2BP is a dual location protein which can be targeted to mitochondria as well as to the plasma membrane, a property that is shared with TMIGD1.

The biological function of TMIGD1 localized at mitochondria of kidney-derived HK-2 cells is unclear. Mitochondria are the major site of ATP production in cells and also a major source of reactive oxygen species (ROS), such as superoxide and hydrogen peroxide [31]. An imbalance between ROS generation and ROS removal by antioxidant defense mechanisms results in oxidative stress. Importantly, mitochondrial dysfunction is a contributing factor in many diseases, and oxidative stress as a result of mitochondrial dysfunction has been described to contribute to the pathogenesis of kidney disease [32, 33]. Intriguingly, TMIGD1 has been shown to play a protective role during H_2_O_2_-induced oxidative stress in cultured HK-2 cells [14]. In vivo, TMIGD1 expression undergoes a biphasic up- and downregulation in two kidney disease models characterized or favoured by excessive ROS generation, i.e. ischemia/reperfusion-induced oxidative stress and experimentally induced hypertension [14]. Our findings of an interaction between TMIGD1 and SYNJ2BP provide a first insight into a possible mechanism through which TMIGD1 could be targeted to mitochondria where it could possibly exert its function in protecting against oxidative stress.

Mitochondria are also involved in cell metabolism and signalling processes [34]. In recent years, accumulating evidence indicates that extracellular proteins as well as plasma membrane-resident proteins are translocated to mitochondria to regulate cellular functions. For example, the epidermal growth factor receptor (EGFR) is translocated to mitochondria where it phosphorylates the cytochrome c oxidase subunit II (CoxII) to reduce CoxII activity and cellular ATP [35]. EGFR translocation to mitochondria can also be triggered by apoptosis inducers and by EGFR kinase inhibitors, which renders cells more resistant to pharmacologically induced apoptosis [36]. In endothelial cells, soluble anti-angiogenic proteins like angiostatin or isthmin are internalised through the endocytic pathway and trafficked to mitochondria through the direct fusion of late endosome (LE) with mitochondria, where they execute apoptosis [37, 38]. Interestingly, SYNJ2BP has been found to mediate the tethering of mitochondria to the rough ER through an interaction that involves its cytosol-exposed PDZ domain and the C-terminal PDZ domain-binding motif of the ER membrane-inserted ribosome-binding protein 1 (RRBP1) [39]. It is therefore possible that increased endocytosis of TMIGD1 in subconfluent cells regulates the trafficking and/or targeting of LE to mitochondria via a TMIGD1 - SYNJ2BP interaction, where it could play a protective role during oxidative stress [14].

The biological function of TMIGD1 at cell-cell contacts is also unknown. TMIGD1 undergoes homophilic interactions and promotes cell aggregation when ectopically expressed in HEK293 cells [14]. These observations support an adhesive function of TMIGD1. Consistent with this, TMIGD1 expression reduces proliferation and cell migration, and increases the barrier properties of HEK293 cells [14]. These findings, together with our observation of a gradual increase in cell-cell contact localization of TMIGD1 with increasing cell density (Fig. 4) suggest that TMIGD1 plays a signalling role that is triggered by trans-homophilic interactions between TMIGD1 molecules on opposing cells, which would be similar to what has been described for members of the JAM family [23]. More work has to be invested to understand the biological role of TMIGD1 in more detail.

## Conclusions

In conclusion, we find that the cell adhesion receptor TMIGD1 can localize to both mitochondria and cell-cell contact sites in renal epithelial cells, and that its localization is regulated by cell confluency. We identify the mitochondrial outer membrane protein SYNJ2BP as interaction partner of TMIGD1, and we find that SYNJ2BP can recruit TMIGD1 to mitochondria. Our findings provide a first insight into a potential molecular basis for the role of TMIGD1 as protector against oxidative stress.

## Methods

### Cell culture and transfections

HK-2 cells (ATCC CRL-2190) were grown in DMEM/F12 (3:1) (Sigma-Aldrich #D5671/ Sigma-Aldrich #51651C) containing 10% FCS, 2 mM glutamine, 100 U/ml penicillin and 100 U/ml streptomycin. HEK293T cells (ATCC CRL-3216) and MDCK II Tet-Off cells (TakaraBio-Clontech, St-Germain-en-Laye, # 630913/631138) were grown in DMEM containing 10% FCS, 2 mM glutamine, 100 U/ml penicillin and 100 U/ml streptomycin. MDCK II Tet-Off cell lines stably expressing human wildtype TMIGD1 (TMIGD1), TMIGD1 lacking the C-terminal PDZ domain-binding motif (TMIGD1/Δ5), a TMIGD1 mutant lacking the membrane-distal Ig-domain (ΔD1-TMIGD1), or a TMIGD1 mutant lacking both the membrane-distal Ig-domain and the PDZ domain-binding motif (ΔD1-TMIGD1/Δ5) were generated by electroporation and subsequent selection by growth in DMEM medium supplemented with 100 μg/ml G418, 1 μg/ml puromycin, 150 μg/ml hygromycin and 50 ng/ml doxycycline. Expression of transgenes was induced by transferring cells into medium lacking doxycycline using tetracycline-free FCS (BD Biosciences) as described [22]. HK-2 cells stably expressing a TMIGD1-specific shRNA (5′-CCAGGCTCAAATGATGTGGTA-3′) under a doxycycline-regulated promoter (TetON) were generated by lentiviral transduction with pInducer10-mir-RUP-PheS (Addgene plasmid #44011 [40];). Expression of shRNAs was induced with 1 μg/ml doxycycline. Transient transfections of HEK293T cells and MDCK cells were performed with Xfect reagent (TakaRaBio-Clontech #631318) and Lipofectamine 2000 reagent (Thermo Fisher Scientific #11668027), respectively, acc. to the manufacturers instructions.

The following plasmid vectors were newly generated: pKE576hyg is a pTRE2hyg (TakaraBio-Clontech)-based plasmid vector that contains the preprotrypsin signal sequence followed by a Flag tag to allow doxycycline-regulated (TetOFF) expression of Flag-tagged constructs. pKE1079 is a pFlag-CMV-1 (Sigma-Aldrich, Munich, Germany)-based plasmid vector that contains EGFP in the multiple cloning site to allow for the expression of Flag-tagged EGFP-constructs.

The following constructs were used. Flag-TMIGD1 constructs in pFlag-CMV-1 (Sigma-Aldrich, Munich, Germany): human full length TMIGD1 without signal peptide (AA 30–262) and human full length TMIGD1 without signal peptide lacking the PDZ domain binding motif (hTMIGD1/Δ5, AA 30–258). Flag-TMIGD1 constructs in pKE576hyg: hTMIGD1 (AA 30–262), hTMIGD1/Δ5 (AA 30–257), hΔD1-TMIGD1 (lacks membrane-distal Ig-like domain, AA 115–262), hΔD1-TMIGD1/Δ5 (AA 115–257). EGFP-TMIGD1 constructs in pKE1079 (EGFP inserted between AA211 and AA212 of TMIGD1): EGFP-hTMIGD1 (AA 30–262). TMIGD1/JAM-A domain swapping constructs in pKE1079: EGFP-TMIGD1-JAM-A (NH_2_-TMIGD1_30–211_-EGFP-JAM-A_231–299_-COOH); EGFP-JAM-A-TMIGD1 (NH_2_-JAM-A_28–230_-EGFP-TMIGD1_211–262_-COOH). EGFP-JAM-A constructs in pKE1079: EGFP-hJAM-A (AA 28–299; EGFP inserted between AA230 and AA231 of JAM-A). GST-TMIGD1 and GST-TMIGD1/Δ5: hTMIGD1 cytoplasmic tail (AA 242–262) and hTMIGD1 cytoplasmic tail lacking the PDZ domain binding motif (AA 242–257), respectively, in pGEX-4 T-1 (GE Healthcare, Solingen, Germany). pBTM116-TMIGD1: cytoplasmic tail of hTMIGD1 (AA 241–262) in yeast-two hybrid vector pBTM116 [41]. Flag-mSynj2bp: murine Synj2bp/Arip2 full length (AA 1–145) in pCS2-pFlag (kindly provided by Dr. Andreas Fischer, DKFZ Heidelberg). Myc-hSYNJ2BP: human SYNJ2BP full length (AA 2–145) in pKE081myc [42].

### Antibodies and reagents

The following antibodies were used in this study: rabbit pAb anti-hSYNJ2BP (Sigma-Aldrich #HPA000866); rabbit pAb anti-TMIGD1 (Sigma-Aldrich #HPA021946); rabbit pAb anti-TMIGD1 (Novus #NBP1–80672); mouse anti-EEA1 (Beckton-Dickinson (BD)-TL #610154); mouse anti-P230 transGolgi (BD-TL #611280); mouse anti-GM130 (BD-TL #610822), mouse mAb anti β-catenin (BD-TL #610456); mouse mAb anti-ZO-1 (BD-TL #610966); mouse mAb anti-α-Tubulin (Sigma-Aldrich, clone B-5-1-2, #T5168); mouse mAb anti-Flag M2 (Sigma-Aldrich #F1804); rabbit pAb anti-Flag (Sigma-Aldrich #F7425); goat pAb anti-Myc (SantaCruz #sc-789G); mouse mAb anti-Myc 9E10 [43]; goat pAb anti GST (GE Healthcare #27–4577-01). Rabbit anti-TMIGD1 pAb Affi1611 was generated by immunizing rabbits with a synthetic peptide (NGKTENYILDTTPGS) coupled via a C-terminal Cys residue to KLH. The serum obtained from immunized rabbits was purified by affinity chromatography using the immobilized peptide as affinity ligand (Biomatik, Wilmington DE, USA). Secondary antibodies and fluorophore-conjugated antibodies: IRDye 800CW Donkey anti-Rabbit IgG (LI-COR Biosciences #926–32,213), IRDye 680CW Donkey anti-mouse IgG (LI-COR Biosciences #926–68,072), Alexa Fluor488 Goat anti-rabbit IgG (Thermo-Fisher #A11034), Alexa Fluor568 Donkey anti-mouse IgG (Thermo-Fisher # A10037). The following reagents were used: FITC-conjugated phytohemagglutinin (PHA)-E (VectorLabs #1111, Biozol, Eching, Germany); PNGase F (Sigma #7367), tunicamycin (Applichem #A2242,0005), doxycycline (Sigma #D9891), collagen type I (rat tail type 1 collagen, Advanced BioMatrix #5163), MitoTracker (MitoTracker Green FM #M-7514, Mitotracker Red CM-H2-XROS, Invitrogen).

### Flow cytometry

MDCK II Tet-Off cell lines stably expressing Flag-TMIGD1 constructs were washed in PBS, incubated for 10 min at 37 °C in PBS / 5 mM EDTA followed by incubation with Accutase® (Sigma Aldrich #A6964) for 10 min at 37 °C. For the analysis of cell surface-localized proteins, cells were incubated with rabbit pAb anti-Flag in CellWASH (BD #349524) containing 5% BSA (1 h, 4 °C), followed by incubation with AlexaF488-conjugated anti-rabbit pAb (45 min, 4 °C). For intracellular stainings, cells were washed, fixed in PBS / 2% PFA, and incubated with primary and secondary antibodies in the presence of 0.5% saponin (Sigma Aldrich #A47036). After washing, cells were resuspended in CellWASH containing 5% BSA at 10^6^ cells per ml and analyzed by flow cytometry using a Guava® easyCyte™ flow cytometer (MerckMillipore). For each sample, 10.000 cells were measured. Results were analysed using the Guava® InCyte™ software (MerckMillipore) and are plotted as histograms.

### Mitochondria labelling with MitoTracker dyes

Labelling of mitochondria was performed using MitoTracker Red CM-H2XRos or MitoTracker Green FM (Invitrogen) acc. to the manufacturer instructions. Exposure to MitoTracker probes (150 μM) was limited to 45 min until cells were washed with PBS and fixed with 4% PFA.

### Yeast two-hybrid screen

Yeast two-hybrid screening experiments were performed essentially as described [42]. Briefly, the *Saccharomyces cerevisiae* reporter strain L40 expressing a fusion protein between LexA and the cytoplasmic tail of TMIGD1 (AA 241–262) was transformed with 250 μg of DNA derived from a day 9.5/10.5 mouse embryo cDNA library [29] according to the method of Schiestl and Gietz [44]. The transformants were grown for 16 h in liquid selective medium lacking tryptophan, leucine (SD-TL) to maintain selection for the bait and the library plasmid, then plated onto synthetic medium lacking tryptophan, histidine, uracil, leucine, and lysine (SD-THULL) in the presence of 1 mM 3-aminotriazole. After 3 days at 30 °C, large colonies were picked and grown for additional 3 days on the same selective medium. Plasmid DNA was isolated from growing colonies using a commercial yeast plasmid isolation kit (DualsystemsBiotech, Schlieren, Switzerland). To segregate the bait plasmid from the library plasmid, yeast DNA was transformed into *E. coli* HB101, and the transformants were grown on M9 minimal medium lacking leucine. Plasmid DNA was then isolated from *E. coli* HB101 followed by sequencing to determine the nucleotide sequence of the inserts.

### Immunoprecipitation and Western blot analysis

For immunoprecipitations, cells were lysed in lysis buffer (50 mM TrisHCl, pH 7.4, 1% (v/v) Nonidet P-40 (NP-40, AppliChem, Darmstadt, Germany), 150 mM NaCl, protease inhibitors (Complete Protease Inhibitor Cocktail; Roche, Indianapolis, IN) and phosphatase inhibitors (PhosSTOP™, Roche, Indianapolis, IN), 2 mM sodium orthovanadate) for 30 min on ice. Postnuclear supernatants were incubated with 3 μg of antibodies coupled to protein A– or protein G–Sepharose beads (GE Healthcare, Solingen, Germany) overnight at 4 °C. Beads were washed five times with lysis buffer, bound proteins were eluted by boiling in SDS-sample buffer/1 mM DTT. Eluted proteins were separated by SDS–PAGE and analyzed by Western blotting with near-infrared fluorescence detection (Odyssey Infrared Imaging System Application Software Version 3.0 and IRDye 800CW-conjugated antibodies; LI-COR Biosciences, Bad Homburg, Germany).

### GST pulldown experiments

In vitro binding experiments were performed with recombinant GST-fusion proteins purified from *E.coli* and immobilized on glutathione-Sepharose 4B beads (Life Technologies). Purification of GST fusion proteins was performed as described [42]. For protein interaction experiments the putative partner protein (prey) was expressed in HEK293T cells by transient transfection. Cells were lysed as described for immunoprecipitations. Lysates were incubated with 3 μg of immobilized GST fusion protein for 2 h at 4 °C under constant agitation. After 5 washing steps in lysis buffer, bound proteins were eluted by boiling for 5 min in SDS sample buffer, subjected to SDS-PAGE and analyzed by Western blotting using prey-specific antibodies.

### Immunocytochemistry and immunohistochemistry

For immunocytochemistry, cells were grown on collagen-coated glass slides. Cells were washed with PBS and fixed with 4% paraformaldehyde (PFA, Sigma Aldrich) for 7 min. To detect intracellular proteins, PFA-fixed cells were incubated with PBS containing 0.2% Triton X-100 for 15 min. Alternatively, cells were fixed with methanol by incubation in icecold methanol for 5 min. Cells were washed with 100 mM glycine in PBS, blocked for 1 h in blocking buffer (PBS, 10% FCS, 0.2% Triton X-100, 0.05% Tween-20, 0.02% BSA) and then incubated with primary antibodies in blocking buffer for 1 h at room temperature (RT) or overnight at 4 °C. After incubation, cells were washed three times with PBS and incubated with fluorochrome (AlexaFluor488, AlexaFluor594 and AlexaFluor647)-conjugated, highly cross-adsorbed secondary antibodies (Invitrogen) for 2 h at RT protected from light. F-Actin was stained using phalloidin-conjugates (FITC, TRITC and AlexaFluor647), DNA was stained with 2,4,diamidino-2-phenylindole (DAPI, Sigma-Aldrich). Samples were washed three times with PBS and mounted in fluorescence mounting medium (Mowiol 4–88, Sigma Aldrich).

For immunohistochemistry, kidney tissue of C57BL/6 J male mice, 12 weeks old, was used. All animals were killed by bleeding (thoracotomy and puncture of the left heart ventricle with subsequent opening of the vena cava) under deep isoflurane anesthesia (1 L O_2_/min; 5% isoflurane). Tissues were fixed in 4% PFA in PBS for 12 h and embedded in paraffin. Paraffin-embedded sections were deparaffinized with xylene and rehydrated through graded alcohols to water. Antigen retrieval was performed by boiling the sections in a pressure cooker for 5 min in TE buffer. A standard Dako EnVison+™ detection kit (Dako, Glostrup, Denmark) was used according to the manufacturers instructions. For immunfluorescence analyses in the kidney, sections were incubated with anti-TMIGD1 antibodies, followed by incubation with fluorochrome-conjugated secondary antibodies and FITC-conjugated PHA-E (dil 1:200), a marker for proximal kidney tubules [25]. The slides were mounted with aquamount after counterstaining with DAPI for 2 min. Images were acquired using an Axio Zeiss microscope (Axiovert 100; Carl Zeiss, Oberkochen, Germany), equipped with a digital camera (AxiocamMRc; Carl Zeiss) with the AxionVisonLE Release 4.7.1 software (Carl Zeiss). Immunofluorescence microscopy of cultured cells was performed using the confocal microscopes LSM 780 and LSM 800 Airyscan (both from Carl Zeiss, Jena, Germany) equipped with the objectives Plan-Apochromat × 40/1.3 oil differential interference contrast and Plan-Apochromat × 63/1.4 oil differential interference contrast (Carl Zeiss). Image processing and quantification was performed using ImageJ, Zen 2 (Blue Edition, Carl Zeiss) and Imaris (Bitplane, Version 9.1.2) software.

### Statistics

For the quantitative analysis of TMIGD1 localization at cell-cell junctions, cells were visually inspected for TMIGD1 immunofluorescence signals. TMIGD1 signals were rated as cell-cell contact-localized when a clear overlap with immunofluorescence signal of cell-cell contact-localized proteins (β-catenin in Figs. 1d, f; ZO-1 in Fig. 4b) at linear cell-cell contact sites was observed. For the quantitative analysis of TMIGD1 signal intensities at cell-cell contacts (Fig. 4b) the ImageJ software was used. Cell-cell contacts were identified as ZO-1-positive regions. Using a mask covering a defined area of 6.75 μm × 6.75 μm the absolute pixel values at ZO-1-positive cell-cell contacts were measured, and the mean values were calculated. For the quantitative analysis of TMIGD1 recruitment to mitochondria by Myc-tagged SYNJ2BP (Fig. 6), cells were visually inspected for the localization of Myc-SYNJ2BP at mitochondria. TMIGD1 signals were rated as mitochondria-localized when a clear overlap with the Myc-SYNJ2BP signal at mitotracker-positive mitochondria was observed. Results are expressed either as arithmetic means ± SEM or ± SD as indicated. To test the normality of data sample distributions, the D’Agostino-Pearson normality test was used. Data were statistically compared using unpaired, two-tailed Student’s *t*-test (cell contacts of stably transfected MDCKII cells), or probed for being statistically different from a fixed value using One sample *t*-test. Statistical analyses were performed using GraphPad Prism version 6 (GraphPad Software, San Diego, CA). *P*-values are indicated as follows: *P < 0.05, **P < 0.01, ***P < 0.001 and ****P < 0.0001.

## Supplementary information

**Additional file 1: Suppl. Fig. 1.** ΔD1-TMIGD1 is retained in the cis-Golgi compartment in the presence of tunicamycin. ΔD1-TMIGD1-expressing MDCKII-TetOFF cells were incubated with tunicamycin as indicated. Cells were fixed with methanol and double-stained with antibodies against the Flag tag to detect ΔD1-TMIGD1 and with either Mitotracker Red to visualize mitochondria (top panels), or with antibodies against GM130 (a cis-Golgi matrix protein) (middle panels) or against p230TG (a peripheral membrane protein associated with the cytosolic face of the trans Golgi network) (bottom panels) to visualize Golgi compartments. In tunicamycin-treated cells, ΔD1-TMIGD1 shows a partial co-localization with Golgi markers but not with Mitotracker Red. Abbreviations: TM, tunicamycin. Scale bars: 10 μm.

## Data Availability

All data generated or analyzed during this study are included in this published article.

## Abbreviations

TMIGD1: Transmembrane and immunoglobulin domain-containing protein 1
JAM-A: Junctional Adhesion Molecule-A
SYNJ2BP: Synaptojanin-2-binding protein
