# Supplementary figures and images for: The mitochondrial outer membrane protein SYNJ2BP interacts with the cell adhesion molecule TMIGD1 and can recruit it to mitochondria
Source: BMC Mol Cell Biol. 2020 Apr 17;21:30. doi: 10.1186/s12860-020-00274-1 (PMC7164261; doi:10.1186/s12860-020-00274-1)

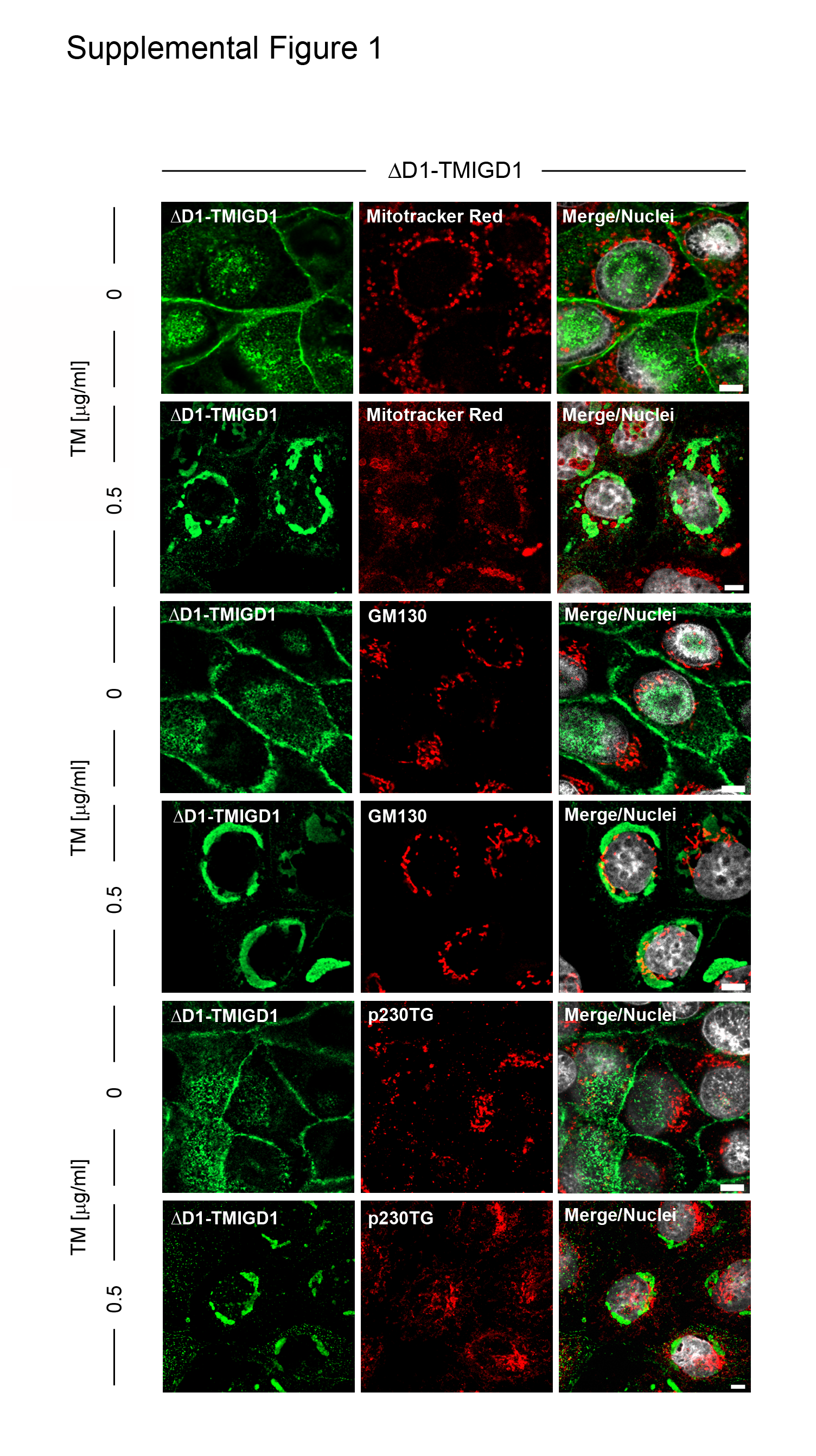

Supplement: Supplementary file 1 — Additional file 1: Suppl. Fig. 1. ΔD1-TMIGD1 is retained in the cis-Golgi compartment in the presence of tunicamycin. ΔD1-TMIGD1-expressing MDCKII-TetOFF cells were incubated with tunicamycin as indicated. Cells were fixed with methanol and double-stained with antibodies against the Flag tag to detect ΔD1-TMIGD1 and with either Mitotracker Red to visualize mitochondria (top panels), or with antibodies against GM130 (a cis-Golgi matrix protein) (middle panels) or against p230TG (a peripheral membrane protein associated with the cytosolic face of the trans Golgi network) (bottom panels) to visualize Golgi compartments. In tunicamycin-treated cells, ΔD1-TMIGD1 shows a partial co-localization with Golgi markers but not with Mitotracker Red. Abbreviations: TM, tunicamycin. Scale bars: 10 μm. [file 12860_2020_274_MOESM1_ESM.tif]
